# The impact of high-speed rail on SO_2_ emissions—based on spatial difference-in-differences analysis

**DOI:** 10.1038/s41598-023-49853-0

**Published:** 2023-12-21

**Authors:** Na Yan, Youshuai Sun, Shanlang Lin, Jingxian Wang, Tuolei Wu

**Affiliations:** 1 School of Economics and Management, Tongji University, Shanghai, 200092 China; 2 Xianda College of Economics and Humanities, Shanghai International Studies University, Shanghai, 200083 China

**Keywords:** Environmental economics, Climate-change mitigation

## Abstract

SO_2_ emissions have brought serious hidden danger to human health and environmental quality, thus hindering sustainable economic development. The development of high-speed rail indirectly has an important impact on SO_2_ emissions through its economic effects. Controlling SO_2_ emissions from the source has increasingly become the focus of many scholars, and it is very important to assess the environmental effects of high-speed rail on SO_2_ emissions reduction. We use the panel data of 285 cities in China from 2007 to 2017, and adopt the spatial Difference-in-Differences model to study the impact of the opening of high-speed rail on SO_2_ emissions. We also introduce an improved spatial DID model that distinguishes neighboring treatment groups and neighboring control groups to test the spatial spillover effect of high-speed rail on neighboring heterogeneous samples. We find that the opening of high-speed rail significantly reduces the city’s SO_2_ emissions through the internal accumulation effect of technological innovation and industrial structure optimization and the urban external interaction mechanism of the cross-regional flow of production factors. Moreover, the spatial spillover effect of the opening of high-speed rail on neighboring cities is significantly positive, especially the spatial spillover effect of HSR on SO_2_ emissions from neighboring cities without HSR. In addition, heterogeneity analysis shows that the effect varies with the different cities’ tiers and income levels. These findings are conducive to accurately assessing the environmental effects of high-speed rail, and provide important policy references for achieving sustainable development and reducing SO_2_ emissions.

## Introduction

With the rapid advancement of urbanization and industrialization, China is facing a serious threat of environmental pollution. In the past few decades, China’s extensive economic growth model has been highly dependent on coal-based fossil energy consumption, and SO_2_ emissions from coal combustion are a major environmental problem that the Chinese government faces^1^. In 2006, China’s SO_2_ emissions reached a historical peak of 25.89 Mt, of which industrial emissions were 22.35 Mt. This not only hinders the sustainable development of China’s economy and environment, but also brings hidden danger to human health. On the one hand, the SO_2_ discharged into the atmosphere interacts with water vapor and metal-suspended particles, which easily form acidic substances, which in turn form acid precipitation and acid rain. This causes a series of serious ecological problems, such as soil and water acidification, climate change, corrosion of building materials, crop loss, etc. On the other hand, a large amount of SO_2_ emissions also has a serious impact on human health, which can lead to respiratory abnormalities, cardiovascular dysfunction, bronchoconstriction and other diseases, and even premature birth and infant death^2,3^. Since then, with the implementation of industrial emission desulfurization projects and the overall promotion of special emission standards for important industries and regions, SO_2_ emissions have been reduced. In 2019, China’s SO_2_ emissions were about 4.57 Mt, of which industrial emissions were about 3.95 Mt. In recent years, China has put a lot of effort into pollution reduction and has made breakthroughs in the reduction of SO_2_ emissions. However, its overall emissions are only second to India and Russia, and it is still one of the countries with the largest SO_2_ emissions in the world^4^. It is still a top priority for government departments to increase efforts to implement efficient and feasible measures for SO_2_ emission reduction. Previous studies have shown that SO_2_ emissions can be attributed to direct fossil fuel consumption in the form of coal and crude oil, such as coal-fired power plants, industrial coal-fired, smelters, oil and gas refining or combustion, and transportation^5^. Controlling SO_2_ emissions from the source has increasingly become the focus of many scholars.

Many scholars have adopted SO_2_ as the dependent variable in empirical models to analyze the path of SO_2_ emission reduction in terms of economic growth, population size, technological innovation, energy consumption, industrial structure, environmental regulation, and transportation^6–11^. In particular, the development of rail transit has reshaped the regional transportation system, and the green and sustainable travel mode has built a comprehensive system of ‘vehicle-oil-road’ integrated emission reduction. In the past ten years, China has implemented large-scale high-speed rail (HSR) construction. According to relevant statistics, China’s HSR operating mileage is close to 40,000 km by the end of 2020, accounting for more than 70% of the global total HSR mileage. In addition, the current highest HSR speed in China has exceeded 350 km per hour, as well as the number of commuters who choose HSR for daily travel has exceeded 2 billion people per year. The rapid development of China’s HSR benefits from government investment and support. One of the reasons is that the government believes that HSR not only serves as an important bridge for economic linkage between cities to promote their economic development, but also promotes the construction of an environment-friendly society with the help of its characteristic advantages in energy saving and emission reduction under the background of high-quality development. Compared with traditional means of transportation, HSR shortens the time and space distance and accelerates the cross-regional flow of various production factors, which inevitably brings a series of economic effects to regional development. The reallocation of production factors among cities leads to the reset of industrial structure and production efficiency, which undoubtedly affects the distribution of urban SO_2_ emissions. However, the opening of HSR may also benefit the center city at the expense of the peripheral cities due to the ‘siphon effect’^12–14^. In particular, the impact of HSR on SO_2_ emissions from its neighboring cities should also be emphasized. Therefore, it is important to comprehensively assess the SO_2_ emission reduction effects of large-scale HSR construction carried out in China from both internal and external city perspectives, and quantitatively analyzing these effects is the theoretical basis for the transition of China’s transportation infrastructure towards green HSR.

In recent years, as global environmental problems have become increasingly serious, scholars have gradually shifted the focus of HSR research from assessing its economic impact to assessing its impact on environmental pollution. On the one hand, HSR has a direct impact on pollution emissions by replacing traditional road vehicles and airplanes. Compared with vehicles such as cars and airplanes, under the premise of the same passenger capacity, HSR consumes relatively low energy and emits comparatively less pollution^15,16^. However, this is not necessarily conducive to environmental improvement for the entire lifecycle of an HSR project^17–19^. For example, the construction process leads to geological damage, habitat loss, and energy consumption^20^. On the other hand, HSR plays an indirect role in environmental pollution through its economic effects. Some studies have shown that the opening of HSR promotes the improvement of the local environment through indirect economic effects such as industrial agglomeration^21^, labor mobility^22^, industrial structure optimization^23,24^, and technological innovation^17,25^. Meanwhile, some studies have claimed that indirect economic effects may also play a role in exacerbating environmental deterioration. For example, the development of HSR promotes industrial transfer and decentralization^26^, and peripheral cities experience a slowdown in economic growth due to a decline in fixed assets^27^, which further increases the pressure on industrial environmental governance in peripheral cities, leading to more serious industrial pollution emissions and energy consumption^28^. The above studies on the environmental impacts of HSR have been assessed, but the conclusions have not yet reached a consensus due to different research perspectives and pollutant indicators. Currently, the existing literature on the environmental effects of HSR is relatively limited, and they focus on the comparison of the relationship between HSR and different pollutant emissions, emphasizing the impact of HSR opening on CO_2_ emission^29,30^. However, the environmental impact of HSR on SO_2_, which seriously endangers air quality and human health, has been ignored.

It is worth mentioning that the studies of Fan et al.^23^, Gao et al.^24^, and Liu et al.^25^ are highly relevant to follow this study. They evaluated the effect of HSR on environmental pollution by using SO_2_ as one of the indicators of environmental pollution, and their results agreed that HSR significantly reduced SO_2_ emissions. Although the existence of regional heterogeneity in this pollution reduction effect was mentioned in their study, as well as analyzed the role of mediating mechanisms of technological innovation and industrial structure, they neglected the spatial spillover effect of HSR network development on SO_2_ emissions. In fact, the stable unit treatment value assumption (SUTVA) may not hold in most cases, and existing empirical articles applying the DID model rarely take this into account. Ignoring spatial correlation leads to the underestimation of standard errors, which exaggerates the significance of the coefficients and leads to serious estimation bias in empirical studies^31,32^. In addition, another literature related to this study is from Li and Guo^17^, who assessed the spatial spillover effect of the pollution abatement effect of HSR, and the conclusion showed that the pollution abatement effect of HSR has a negative spatial spillover effect. In this article, SO_2_ emission is also only used as one of the indicators of pollution emission, and only the spatial spillover of neighboring areas with access to the HSR network is considered to be evaluated, ignoring the spatial spillover effect of the neighboring cities that are not connected to the HSR network. Therefore, we focus on the impact of HSR on urban SO_2_ emissions and its wider spatial spillovers, expecting to complement existing studies. And we try to clarify the following questions: how does HSR affect urban SO_2_ emissions? Does the SO_2_ emission reduction of HSR come at the expense of environmental pollution in other areas? In particular, the heterogeneous spatial spillover effects of the SO_2_ abatement effect of HSR on the neighboring cities that do not have HSR and those that do have HSR are worthy of attention.

We use a panel data set of 285 cities in China from 2007 to 2017, and apply multiple econometric methods such as the spatial DID model, the instrumental variable method, and the mediation model to evaluate the environmental effects of the HSR opening on SO_2_ emissions. Specifically, this article has made three main contributions. (1) Although SO_2_ emissions are mentioned in very few studies^24,33,34^, it is usually used as a part of the factors in measuring environmental pollution or as a control variable of the model to simply explain the statistical significance, without in-depth analysis and research on the mechanism behind it. We assess the environmental impacts of the opening of HSR on SO_2_ emissions from the perspective of the city’s internal accumulation effect of technological innovation and industrial structure optimization and the urban external interaction mechanism of the cross-regional flow of production factors. This provides policy insights for China to coordinate the sustainable development of the economy and environment in the ‘new normal’ development stage. (2) The current study ignores the spatial spillover effect of the HSR network, which leads to bias in the evaluation of HSR’s effect on SO_2_ emission reduction. Therefore, this paper considers the spatial externality of the HSR network on SO_2_ emissions and uses spatial measurement models to evaluate the spatial spillover effect of HSR on SO_2_ emissions. (3) The spatial spillover effect is weighed by two opposing ‘spillover effects’, the positive impact of factor agglomeration and the negative impact of factor mobility, and how to identify and compare these two spillover effects remains a challenge for current research on the spatial spillover effect of pollution emissions. We refer to Yan et al.^35^ who used an advanced spatial DID model to distinguish the spillover effects of HSR on the surrounding treatment and control groups. Compared to the traditional spatial DID model that only emphasizes the spatial spillover effect on the treatment group, this study combines the spatial econometric model with the DID approach using the advanced SDID specification, which not only captures the unobserved spatial correlation of heterogeneous spatial units, but also identifies the two aforementioned spatial spillover effects and comparatively analyzes them.

## Theory, data, and methods

### Theoretical analysis

Based on the characteristics of cross-regional mobility of production factors driven by the development of the HSR network, this study explains the mechanism of high-speed rail affecting urban SO_2_ emissions in terms of both intra-city accumulation and extra-city interaction.

#### Intra-city accumulation

Firstly, the HSR network promotes SO_2_ emission reduction by enhancing technological innovation. On the one hand, HSR is conducive to intercity travel and face-to-face communication, so that various elements can achieve better matching and interaction at the regional level, generating knowledge spillover and diffusion^36^, thereby enhancing urban innovation. Although the current Internet technology is very advanced, such information technology cannot perfectly replace the face-to-face interaction of these highly skilled talents^37^. On the other hand, HSR is famous for its features of high speed, convenience, and comfort, which are especially favored by high-skilled individuals. HSR reduces commuting costs and expands the matching radius for employers looking for high-skilled individuals, and some high-skilled individuals tend to move their residences to cities along high-speed rail lines. HSR network development has greatly enhanced the attraction of cities to high-quality talent, accelerated the concentration of talent, and promoted the development of low-carbon technologies. Levinson^38^ studied the relationship between technological advancement and pollution emissions in the United States and found that technological advancement reduced SO_2_ emissions by 39%. In particular, it promotes green technological innovation. Huang & Wang^39^ believed that HSR promotes the flow of innovative factors, which in turn helps to improve the efficiency of green innovation. Therefore, we believe that the HSR opening affects SO_2_ emissions through the mechanism of the technological innovation effect.

Secondly, the HSR network promotes SO_2_ emission reduction by optimizing the industrial structure. On the one hand, the opening of HSR has created many opportunities for the development of the service industry, especially the service industries such as catering, accommodation, and entertainment near the HSR station have been vigorously developed due to the large flow of people^40^. The development of the service industry increases local environmental regulations and land prices, while crowding out industries with high pollution emissions^41^, and SO_2_ emissions decrease as the proportion of the tertiary industry increases^23^. Meanwhile, the development of HSR has also promoted the rise and development of e-commerce, information technology, consulting, and other service industries in cities along the line. The development of modern service industries is usually an important way to optimize the industrial structure^42^, which helps to reduce SO_2_ emissions^43^. On the other hand, with the network development of HSR, the connectivity and accessibility between cities have been improved, which reduces the transportation cost of production materials and commuting time. As a result, it allocates resources more reasonably and promotes industrial transformation and upgrading. Some central cities with developed HSR attract middle and high-end industries from other cities through the industrial agglomeration effect. Adjusting and optimizing the industrial structure is the key to controlling the total emissions of regional pollutants and ensuring environmental quality. HSR opening promotes the upgrading and optimization of industrial structures, thereby reducing SO_2_ emissions. Therefore, HSR opening affects SO_2_ emissions through the mechanism of the industrial structure effect.

Therefore, Hypothesis 1 is put forward:

**Hypothesis 1**: *Compared with cities without high-speed rail, cities with high-speed rail have achieved internal accumulation through technological innovation and industrial structure optimization, thereby reducing SO*_*2*_* emissions*.

##### Extra-city interaction

Further, the development of HSR networks has greatly reduced transportation costs between cities^21,44,45^, and factors of production have been transferred across regions through HSR networks^46^. Production factors that are highly related to industrial pollution are also flowing across regions between cities connected by HSR, especially in adjacent cities. Zhou and Zhang^14^ believed that HSR has affected China’s industrial development by promoting spillover effects and siphon effects in different industries. Then, the reallocation of regional industries by high-speed rail development has led to a regional transfer of industrial pollution^17^, and HSR development has generated spatial spillover effects outside the city through interaction with neighboring cities. That is, the opening of HSR not only has an important impact on local SO_2_ emissions, but also has a certain impact on SO_2_ emissions in surrounding areas.

Therefore, Hypothesis 2 is put forward:

**Hypothesis 2**: *The impact of HSR on SO*_*2*_* emissions has significant spatial spillover effects*.

Finally, the spatial spillover effect of HSR on SO_2_ emissions comes from two aspects: the positive impact of factor agglomeration and the negative impact of factor flow^47^. On the one hand, HSR is conducive to the cross-regional flow of skilled managers for learning and communication, reducing the cost of technology research and development, which in turn affects the application of cleaner technologies in production and increases the spatial spillover of cleaner technologies through imitation and learning effects. For example, Huang et al.^48^ showed that urban road infrastructure has no direct impact on industrial pollution, but does affect industrial pollution in neighboring cities through the spatial spillover effect of industrial agglomeration. On the other hand, HSR promotes the transfer of production resources to cities along HSR, and further flows from small and medium-sized cities to central cities, which results in the reallocation of resources and regional polarization^49^. Due to the relatively strong siphoning effect, modern service industries are more inclined to cluster in the central cities along the HSR line. While other cities along the HSR line have taken over more pollution-related enterprises transferred from the central cities, and HSR may further aggravate pollution emissions in these cities. In addition, in order to reduce operating costs, HSR has prompted manufacturing firms to set up headquarters or R&D centers in central cities and move production departments to peripheral cities^50,51^, which has facilitated the diffusion of population and production resources from the core to peripheral cities along the HSR line^52^. Based on the new economic geography theory, the spatial sorting effect enables efficient enterprises to gather in central cities, while inefficient enterprises move to peripheral areas^53^, which undoubtedly increases pollution emissions in peripheral areas along the HSR line.

There is spatial spillover heterogeneity in SO_2_ emissions from surrounding cities with HSR and cities without HSR. In other words, the concentration of production factors promotes economies of scale and knowledge spillovers, which to a certain extent helps to improve the absorption of advanced technologies in surrounding areas, thereby improving production efficiency and reducing emissions. In addition, with the expansion of production scale, the production departments of manufacturing enterprises have moved to surrounding cities along HSR lines, especially the production of heavy industries characterized by high pollution emissions, which exacerbated SO_2_ pollution emissions in neighboring cities in the short term.

Therefore, Hypothesis 3 is put forward:

**Hypothesis 3**: *The spatial spillover effect of HSR on SO*_*2*_* emissions from neighboring cities has heterogeneous spatial spillover characteristics in the neighboring control group and neighboring treatment group.*

### Research data

#### Variable description

Based on the data from 285 cities during 2007–2017, this article conducts an empirical study of the environmental effects of HSR on SO_2_ emission reduction. To ensure data reliability, we use the mean imputation method to fill in some missing and invalid data, and eliminate those city samples with serious incomplete data, such as Lhasa, Danzhou, and Sansha.

**Figure 1:**
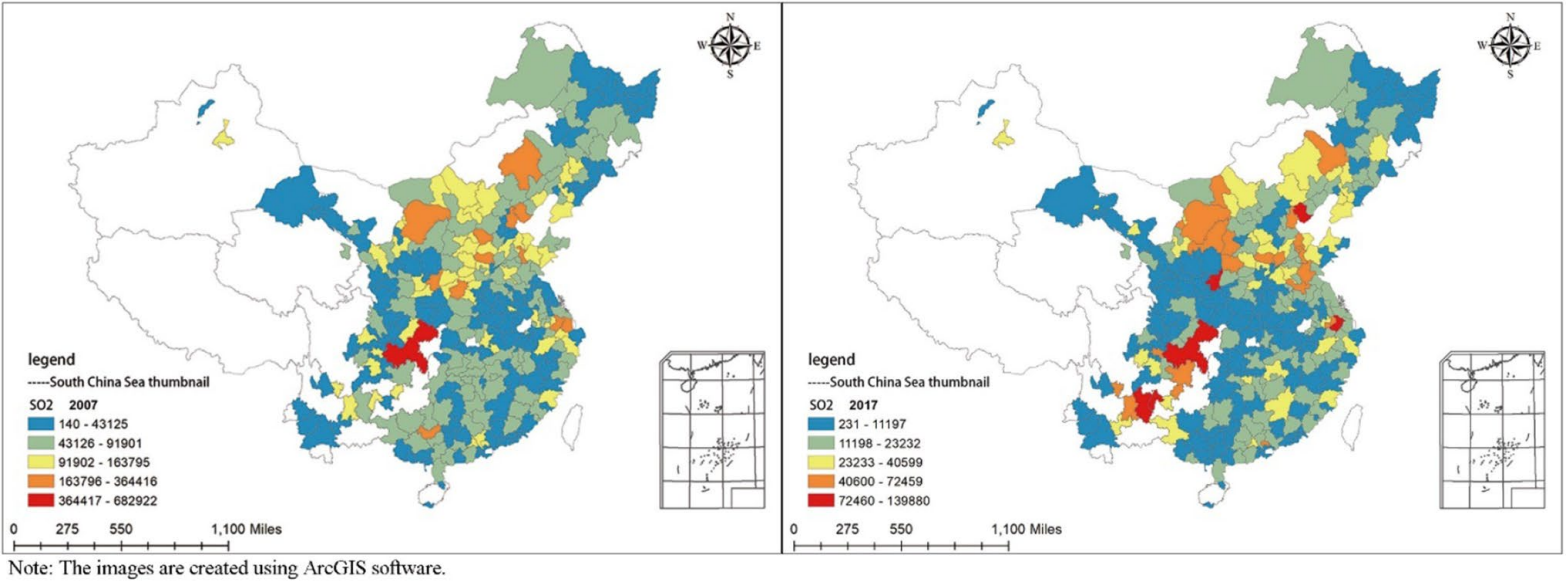
City distribution of SO_2_ emissions in China in 2007 and 2017.

**Explained variable (SO**_**2**_**):** This article adopts China’s city industrial SO_2_ emissions as the core explained variable. Figure 1 is a schematic diagram of the city’s SO_2_ emission spatial distribution in 2007 and 2017 drawn by ArcGIS 10.7. The maximum SO_2_ emissions of Chinese cities dropped from 682,922 tons in 2007 to 139,880 tons in 2017, which shows that SO_2_ emissions have been well controlled. However, the spatial distribution characteristics of SO_2_ emissions shown in Fig. 1 show that there are obvious regional differences. SO_2_ emissions from cities in southwestern and northern China are significantly higher than those in eastern China.

**Explanatory Variables (HSR):** We use a dummy variable to represent the HSR variable, that is, Whether the city has opened HSR or not. If the HSR has been opened this year, the HSR is equal to 1, otherwise, it is 0.

**Control variables:** Studies have shown that variables including economic development, residents’ income, science and technology, investment in fixed assets, and openness are important causes affecting environmental pollution^54,55^. In order to reduce the interference of other influencing factors of SO_2_, this article controls the actual per capita GDP (PGDP), the actual urban residents’ income (Inco), the share of technology in fiscal spending (Scie), the electricity consumption (Elec), and the actual use of foreign capital (FDI). In addition, to eliminate the influence of other transportation infrastructures, this paper also controls the air passenger volume (Avi) in the regression model. Finally, considering the non-linear impact of economic growth on environmental pollution, that is, the Environmental Kuznets Curve (EKC) may exist. Therefore, the square term of per capita GDP (PGDP2) is also included in the econometric model. All control variables are treated logarithmically.

**Mechanism variables:** Through the above analysis, we explain the environmental effects of HSR opening on SO_2_ emission reduction from the two transmission mechanisms of technological innovation (TInno), and industrial structure (IStru). Specifically, we refer to the previous studies to use the number of patent grants to measure the effect of technological innovation^56–58^, and refer to Gu et al.^59^ and Yang et al.^60^ to use the rationalization index of industrial structure to measure the effect of industrial structure, the formula is $L = \mathop \sum \limits_{i = 1}^{n} \left( {\frac{{Y_{i} }}{Y}} \right){\text{ln}}\left( {\frac{{Y_{i} /L_{i} }}{Y/L}} \right)$.

#### Data sources

The data relating to HSR is from the national ‘Medium and Long-term Railway Network Planning’ and ‘China Railway Yearbook’. The data about PGDP, Income, Science, FDI, Avi, Elec, patent, and related variables come from the collection and arrangement of data in the ‘Chinese City Statistical Yearbook’ and ‘Chinese City National Economic and Social Development Statistical Bulletin’.

### Research methods

The difference-in-differences (DID) model is often widely used to evaluate the effectiveness of a certain policy implementation. Many scholars regard the opening of high-speed rail as a quasi-natural experiment and use the multi-period DID method of formula (1) to evaluate the economic impact of HSR opening. Those cities that opened HSR during the period 2007–2017 are used as the treatment group, while the remaining cities that have not opened HSR are used as the control group.

1 $$ Y_{it} = \alpha + \beta HSR_{it} + \varphi Z_{it} + \delta_{i} + \lambda_{t} + \varepsilon_{it} $$

$Y_{it}$ is the SO_2_ emission of city ${ }i$ in year $t$, the key explanatory variable $HSR_{it}$ is a dummy variable, which measures whether city $i$ opened HSR or not in year $t$, the matrix $Z_{it}$ represents the control variable, and $\delta_{i}$ and $\lambda_{t}$ are time fixed effects and city fixed effects, respectively. $\varepsilon_{it}$ is an error term, and $\alpha$ is the constant.

For the DID model, a classical assumption is the Stability of Individual Treatment Effects Assumption (SUTVA). However, SUTVA no longer holds when there is a correlation between different spatial units. As mentioned in the analysis in the previous section, China’s HSR network has rapidly expanded in the past decade, and a large number of studies have confirmed that the network traffic infrastructure has a spatial spillover effect^32^. That is, the network structure characteristics of HSR not only affect local economic and social activities, but also have cross-regional effects on surrounding areas. Therefore, SUTVA is not valid here, and if the issue of spatial correlation is ignored in the empirical testing process, the research results may be biased. The spatial econometrics model examines the interaction effect of the explained variable between the time series and the spatial location^61^. We combine the DID estimation method with spatial econometric modeling to assess the SO_2_ abatement effect of HSR using the spatial Difference-in-Differences mode (SDID). At present, three spatial econometrics models are currently applied in related studies, namely the spatial autocorrelation model (SAR), the spatial error model (SEM), and the spatial Durbin model (SDM), and the most appropriate spatial model is usually chosen according to the source of spatial dependence^62,63^. Since existing literature has proved that China’s HSR infrastructure has spatial spillover effects on economic and social development due to its network distribution^64^, we use the spatial Durbin model (SDM) to test the spatial spillover effects of HSR on SO_2_ emissions. It is worth noting that in order to avoid redundancy and multicollinearity of explanatory variables in the model, this study refers to Yan et al.^35^ to introduce only the spatial lag term of HSR into the spatial Durbin model. Therefore, we construct the following spatial econometrics model.

2 $$ Y_{it} = \alpha + \beta HSR_{it} + \gamma WHSR_{it} + \rho WY_{it} + \varphi Z_{it} + \delta_{i} + \lambda_{t} + \varepsilon_{it} ,{ }\varepsilon_{it} \sim N\left( {0,\sigma^{2} I} \right) $$

where, W in formula (2) is the spatial weight matrix, represented by a row-standardized binary spatial weight matrix. If there are n adjacent cities in a certain city, then the elements of these adjacent cities are all set to 1/n, and the values of the remaining cities are set to 0. ρ refers to the spatial coefficient.

$\gamma WHSR_{it}$ represents the average spillover effect of HSR opening on neighboring cities, but the spillover effect between neighboring treatment groups and control groups may be different. we refer to the methods of Chagas et al.^65^ and Yan et al.^35^ to measure the spillover effects on neighboring treatment and control groups respectively by decomposing the spatial weight matrix. The empirical model is shown in formula (3).

3 $$ Y_{it} = \alpha + \beta HSR_{it} + \theta_{1} W_{T,T} HSR_{it} + \theta_{2} W_{NT,T} HSR_{it} + \rho WY_{it} + \varphi Z_{it} + \delta_{i} + \lambda_{t} + \varepsilon_{it} ,{ }\varepsilon_{it} \sim N\left( {0,\sigma^{2} I} \right) $$

where $\theta_{1} W_{T,T} HSR_{it}$ represents the spillover effect of HSR on surrounding cities with HSR, while $\theta_{2} W_{NT,T} HSR_{it}$ represents the spillover effect of HSR on surrounding cities without HSR.

## Results

### Baseline regression

The simulation results based on the traditional DID model of formula (1) are shown in column (1) of Table 1. It can be found that the coefficient of HSR is negative, which to a certain extent explains the negative correlation between the HSR opening and SO_2_ emissions. However, the coefficient is not significant, which may be due to the defective baseline modeling setup, such as heterogeneous treatment effects, endogeneity, and spatial autocorrelation. Next, we further improve the model to address the above issues.

**Table 1:** Effect of HSR on SO_2_ emission reduction.

Variables	DID	IV-2SLS		SDID	Decomposition of the SDM		
	SO_2_	HSR	SO_2_	SO_2_	**SO**_**2**_		
					Direct	Indirect	Total
	(1)	(2)	(3)	(4)	(5)	(6)	(7)
HSR	− 0.041		− 0.411*	− 0.125***	-0.141***	− 2.893***	− 3.034***
	(0.035)		(0.206)	(0.040)	(0.042)	(0.681)	(0.688)
Rail1962		2.435***					
		(0.502)					
Ruggedness		− 0.228***					
		(0.050)					
W*HSR				− 1.018***			
				(0.179)			
rho				0.617***			
				(0.054)			
F-statistic		41.42					
R-squared	0.547	0.428	0.428	0.156	0.156	0.156	0.156
Control variables	YES	YES	YES	YES	YES	YES	YES
Time Fixed	YES	YES	YES	YES	YES	YES	YES
City Fixed	YES	YES	YES	YES	YES	YES	YES
N	3135	3135	3135	3135	3135	3135	3135
did_multiplegt	− 0.020*						
	(0.012)						

Note: Standard errors clustered at the city level are in parentheses in columns (1)-(3), robust standard errors are in parentheses in columns (4). The star mark represents its significance level, and ***, **, * indicate the significance levels at l%, 5%, and l0%, respectively. The above table only displays the regression results for the main explanatory variables, if you need the complete results for all coefficients please request them from the corresponding author, the following tables are similar.

Firstly, when there are heterogeneous treatment effects, the two-way fixed-effects model described above may lead to a negative weighting, which leads to biased estimated coefficients^66–69^. We test for possible heterogeneous treatment effects in the baseline regression based on the method of De Chaisemartin and D’Haultfoeuille^68,70^. Specifically, the ‘Twowayfeweights’ code is applied to test for possible heterogeneity in treatment effects. The results show that among all 1043 weights, 888 weights are positive and 155 weights are negative, with the proportion of negative weights being only 14.86%, which indicates that the heterogeneous treatment effects may have a certain impact on the results of the baseline regression. Thus we use the heterogeneous robust DID estimator ‘did_multiplegt’ proposed by De Chaisemartin and D’Haultfoeuille^68,70^ to further demonstrate the emission reduction effect of HSR. As shown in the last row of column (1), the coefficient of HSR is significantly negative, that is, the opening of HSR has a significant emission reduction effect on urban SO_2_ emissions after taking into account the heterogeneous treatment effect.

Secondly, due to the influence of factors such as measurement bias, omitted variables and reverse causality, the potential interference of endogenous problems on the estimated results cannot be ignored. Instrumental variable methods are often widely used to alleviate endogeneity problems. In research on transportation infrastructure, geographical feature information^71^ and historical transportation endowment^72,73^ are widely used as instrumental variables to deal with the endogenous problems of transportation infrastructure. Therefore, this paper uses the geographical ruggedness index and the railway density in 1962 as the instrumental variables for the HSR opening to test the endogeneity. On the one hand, the layout of the HSR depends on the cost of geographical development, and the ruggedness index calculated from the difference in altitude fluctuation in the city is the main factor affecting the cost of geographical development. Simultaneously, HSR is the same as the factors investigated in the construction of ordinary railways in any era. Historical railway construction is highly related to the site selection of current HSR stations. On the other hand, geographic features and historical rail are irrelevant to current SO_2_ emissions. It is worth noting that the above two instrumental variables are cross-sectional data. We refer to Duflo and Pande^74^ to multiply the instrumental variable with the time trend item to construct a set of time-varying instrumental variables. Lastly, the two-stage least squares method was applied to carry out the endogeneity test, and the results are shown in columns (2) and (3) of Table 1. Column (2) is the result of the first stage of instrumental variables, which shows that the value of the F statistic is higher than 10, indicating that the selected instrumental variable is related to an endogenous explanatory variable, which also confirms the rationality of the selected instrumental variables. Column (3) is the result of its second stage, and the coefficient of HSR shows that it is significantly negative at the 1% level, thus verifying that the opening of HSR can suppress urban SO_2_ emissions. However, we found that the coefficient of HSR opening in the instrumental variable test is greater than its coefficient in the baseline regression, indicating that potential endogenous problems may underestimate the effect of HSR opening on SO_2_ emissions reduction to a certain extent.

Further, we also focus on the spatial spillover effect and empirically estimate the SO_2_ abatement effect of HSR using the formula (3), and column (4) is the estimated result of the SDID model. The coefficient of W*HSR is significantly negative at the 1% level, indicating that the spatial spillover effect of HSR is obvious. That is, the opening of HSR alleviates SO_2_ emissions in surrounding cities. Meanwhile, the coefficient of *rho* is significantly positive, indicating that the SO_2_ emission reduction effect of HSR has a significant positive spillover effect on surrounding areas. In addition, when we consider the spatial effect, HSR opening still has a significant environmental effect on SO_2_ emissions reduction, and this effect is stronger compared with the baseline model after solving the endogeneity problem, this impact is weaker. This shows that if the spatial spillover effect of HSR is ignored, the traditional DID model overestimates the role of HSR in SO_2_ emission reduction^31^. In order to explore the marginal effects of the regression coefficients in the spatial model, we decomposed the spatial Durbin model to estimate the direct, indirect, and total effects of HSR on SO_2_ emissions^75^, and the results are shown in columns (5)–(7). The regression coefficients for the direct effect of SDM are inconsistent with column (4) due to feedback effects. That is, the feedback effect refers to changes in explanatory variables within a region, which cause responses in neighboring regions and then return to the region through spatial spillovers. The direct effect shows that HSR significantly reduces local SO_2_ emissions at the 1% level, with a 14.1% reduction in SO_2_. The indirect effect reflects that the coefficient of HSR is significantly negative at the 1% level, a finding that suggests that there is indeed a spatial spillover of the emission reduction effect of HSR. That is, the positive externality of HSR opening also has a significant mitigating effect on SO_2_ emissions in the neighboring areas, and the indirect SO_2_ abatement effect of HSR is significantly larger than the direct abatement effect.

### Robustness test

**Figure 2:**
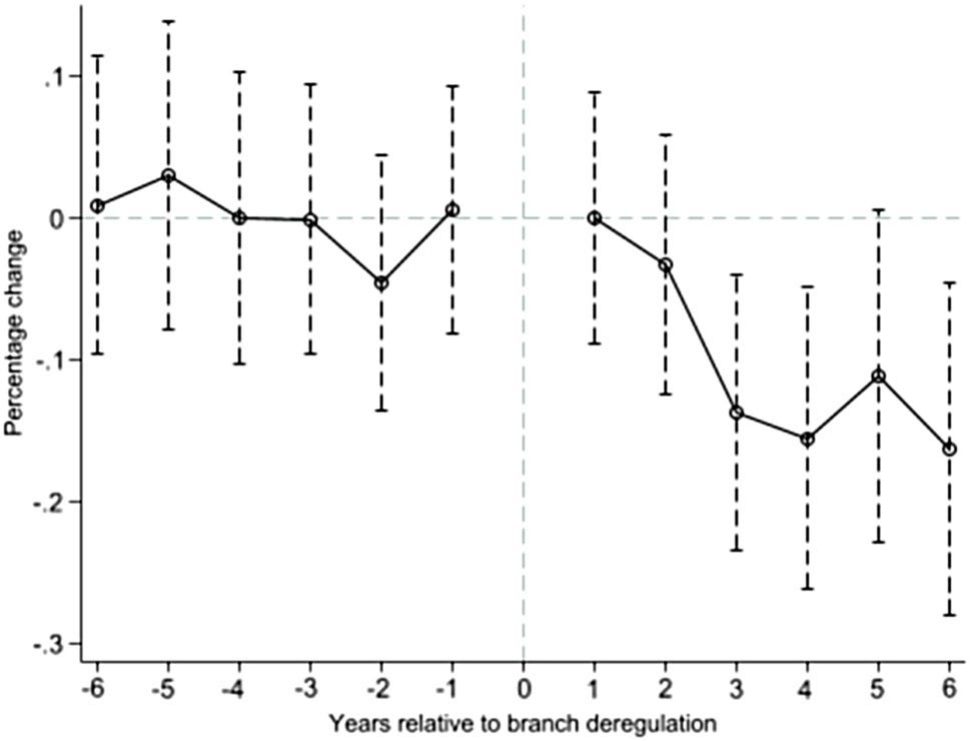
Multi-period DID dynamic effect test chart.

The SDID model further considers the influence of spatial correlation factors based on the DID model. Therefore, we also need to meet the prerequisite for using the DID model, that is, to satisfy the ‘parallel trend hypothesis’ test. It is assumed that the characteristics and trends held by the treatment group are consistent with those of the control group before the policy, and whether the cities of the treatment group have opened HSR is the gap between the two groups. Compared with the control group, the trend change of SO_2_ emissions in the treatment group is mainly from the impact of HSR opening. Therefore, this article adopts the dynamic DID model for the parallel trend test, as shown in Fig. 2. In the horizontal coordinate axis, ‘0’ represents the base period for the opening of HSR, ‘− 6’, ‘− 5’, ‘− 4’, ‘− 3’, ‘− 2’ and ‘− 1’ respectively represent 6, 5, 4, 3, 2 and 1 years before HSR opening. Similarly, ‘1’, ‘2’, ‘3’, ‘4’, ‘5’, and ‘6’ respectively refer to the first year, the second year, the third year, the fourth year, and the fifth year and the sixth year after HSR opening. Figure 2 shows that the influence coefficients of HSR are insignificant before HSR opening, while the coefficient changes from positive to negative, and becomes significant after the second year of opening, which shows that the parallel trend is satisfied, and the emission reduction effect has hysteresis characteristics.

We also performed robustness tests in Table 2, such as changing the sample period, Bilateral censoring treatment at 1% level, placebo test, and changing the explanatory variable and spatial matrix.

**Table 2:** Robustness test of the environmental effects of HSR on SO_2_ emission reduction.

Variables	Change the sample period	Bilateral censoring at the 1% level	Placebo test	Change explanatory variable	Change spatial matrix
	SO_2_				
	(1)	(2)	(3)	(4)	(5)
HSR	− 0.101*	− 0.078**			− 0.120***
	(0.057)	(0.036)			(0.041)
HSR_fault			− 0.008		
			(0.027)		
HSR_station				− 0.008*	
				(0.005)	
W*HSR	− 0.460*	− 0.877***	0.462	− 0.327***	− 1.215***
	(0.272)	(0.159)	(0.295)	(0.046)	(0.328)
rho	0.586***	0.582***	0.324***	0.563***	0.814***
	(0.070)	(0.053)	(0.085)	(0.057)	(0.049)
LR_Direct					
HSR	− 0.106*	− 0.090**	0.007	− 0.012*	− 0.151***
	(0.059)	(0.037)	(0.027)	(0.007)	(0.044)
LR_Indirect					
HSR	− 1.269*	− 2.219***	− 0.344	− 0.762***	− 7.645**
	(0.771)	(0.509)	(0.302)	(0.141)	(3.605)
LR_Total					
HSR	− 1.375*	− 2.309***	− 0.336	− 0.774***	− 7.796**
	(0.777)	(0.514)	(0.302)	(0.143)	(3.618)
R-squared	0.266	0.150	0.080	0.245	0.118
Control variables	YES	YES	YES	YES	YES
Time Fixed	YES	YES	YES	YES	YES
City Fixed	YES	YES	YES	YES	YES
N	1995	3135	3135	3135	3135

Note: Robust standard errors are in parentheses. The star mark represents its significance level, and ***, **, * indicate the significance levels at l%, 5%, and l0%, respectively.

Firstly, considering that the HSR openings in the sample cities were mainly concentrated in the period from 2008 to 2013, we excluded the samples from 2014 to 2017. HSR is still significantly negative in column (1), and the spatial spillover effect is significant. Secondly, considering that the outliers in a large sample produce biased estimates in the regression, and therefore affect the actual results. We carry out a bilateral censoring process at the 1% level for the explained variable. We found that HSR in column (3) still shows a negative correlation to SO_2_ emissions, and the spatial spillover effect is significant. Thirdly, in order to eliminate the influence of SO_2_ emissions caused by other random factors, we refer to Catalini et al.^37^ and conduct the placebo test in column (4). After applying the randomly generating experimental groups to regenerate the explanatory variable of HSR opening (HSR_fault), the coefficient of HSR_fault and W*HSR are not significant, indicating that the randomly generated HSR opening variables cannot affect the SO_2_ emissions. Fourth, considering that the estimation of the multi-period DID model by two-way fixed effects may be biased, we performed a robustness test by changing the estimation model. Specifically, using the continuous variable of the number of HSR stations instead of the dummy variable of HSR opening in column (6) to re-estimate the model. The results show that the coefficient of HSR is significantly negative, and the spatial spillover effect is significant. Lastly, considering that there are samples with no neighboring cities in the selected 285 cities, some cities have multiple neighboring cities, and some cities have only one neighboring city, this may cause estimation bias in the results to a certain extent. Therefore, we also retested the SDM model using the geographic distance matrix to further test the robustness, and the results are shown in columns (5). The results show that the coefficients of ρ and W*HSR are both significant at the 1% level, confirming that SO_2_ emissions have a positive spatial spillover effect on surrounding cities. In addition, from the results of the SBM spatial model decomposition, all models in the robustness test reflect the significant emission reduction effect of HSR on SO_2_, and the indirect emission reduction effect of HSR on SO_2_ is larger than its direct emission reduction effect.

In summary, the above results further verify the conclusion that the opening of HSR can alleviate SO_2_ emissions and that HSR has a spatial spillover effect on SO_2_ emission reduction in surrounding cities. That is, Hypothesis 2 is verified.

## Discussion

### Mechanism inspection

In order to further explore the transmission mechanism by which HSR promotes SO_2_ emissions reduction, we use the mediation effect model and empirical formula (3) to test the mechanism of the intra-city accumulation effect and the external interactive effect of HSR on SO_2_ emission reduction. The results are shown in Table 3.

**Table 3:** Mechanism analysis of the environmental effects of HSR on SO_2_ emission reduction.

Variables	Baseline Model	Technological innovation		Industrial structure		Extra-city interaction
	SO_2_	TInno	SO_2_	IStru	SO_2_	SO_2_
	(1)	(2)	(3)	(4)	(5)	(6)
LR_Direct						
HSR	− 0.141***	1.212***	− 0.137***	0.011*	− 0.135	
	(0.042)	(0.293)	(0.041)	(0.006)	(0.106)	
Mechanism (*TInno* or *IStru*)			− 0.003*		− 0.397**	
			(0.001)		(0.183)	
LR_Indirect						
HSR	− 2.893***	1.836*	− 2.868***	0.348**	− 2.007	
	(0.681)	(1.075)	(0.661)	(0.164)	(1.813)	
Mechanism (*TInno* or *IStru*)			− 0.006*		− 0.757*	
			(0.003)		(0.426)	
LR_Total						
HSR	− 3.034***	3.048***	− 3.005***	0.358**	− 2.142	
	(0.688)	(1.057)	(0.662)	(0.165)	(1.767)	
Mechanism (*TInno* or *IStru*)			− 0.009*		− 1.155*	
			(0.005)		(0.648)	
${W}_{T,T}$ HSR						− 0.015***
						(0.005)
${W}_{NT,T}$ HSR						− 0.016**
						(0.008)
rho	0.617***	− 0.382***	0.615***	0.860***	0.314***	0.473***
	(0.054)	(0.093)	(0.054)	(0.024)	(0.086)	(0.017)
R-squared	0.156	0.528	0.152	0.231	0.107	0.112
Control variables	YES	YES	YES	YES	YES	YES
Time Fixed	YES	YES	YES	YES	YES	YES
City Fixed	YES	YES	YES	YES	YES	YES
N	3135	3135	3135	3135	3135	3135

Note: Robust standard errors are in parentheses. The star mark represents its significance level, and ***, **, * indicate the significance levels at l%, 5%, and l0%, respectively.

Columns (1)–(5) test the intermediary mechanism effect of HSR improving urban technological innovation and industrial structure, thereby affecting SO_2_ emissions. In order to facilitate the comparison of coefficients between models to further identify the mediating effects, only the coefficient results of the decomposition effects of the SBM model are shown in the table. The coefficient of HSR in columns (2) and (4) are significantly positive at least at the 10% level, indicating that HSR promotes technological innovation, and speeds up the upgrading and optimization of industrial structures in both direct and indirect effects. At the same time, the coefficients of mechanism variables including *TInno*, and *IStru* in columns (3) and (5) are all significantly negative. Furthermore, by comparing with the coefficient of HSR in column (1), we find that the HSR coefficient sizes in columns (3) and (5) become smaller, and the HSR in column (5) is not significant. Therefore, it shows that the environmental effect of HSR opening on SO_2_ emission reduction can be achieved through technological innovation and industrial structure. Technological innovation shows a partial intermediary effect, while industrial structure optimization shows a complete intermediary effect. Hypothesis 1 is verified.

Column (6) tests whether the SO_2_ emission reduction effect of HSR has heterogeneous spatial spillovers to surrounding treatment group cities and control group cities by using the formula (3). We find that the coefficients of $W_{NT,T}$ HSR and $W_{T,T}$ HSR are both significantly negative, and the former is slightly smaller than the size of the latter. This shows that the opening of HSR has a positive spatial spillover effect on both neighboring cities with and without HSR, that is, the opening of HSR promotes SO_2_ emission reduction in neighboring cities. However, the spatial spillover intensity of this emission reduction effect has heterogeneous characteristics in the two types of surrounding cities. The spatial spillover effect of the SO_2_ emission reduction effect of HSR on neighboring cities without high-speed rail is slightly greater than that on neighboring cities with HSR. Hypothesis 3 is verified.

This may be because the positive effects such as factor accumulation brought by HSR are the only way to affect neighboring cities without HSR. As for the neighboring cities with HSR opening, the industrial transfer and siphoning effect inevitably exacerbate the SO_2_ emission, but in general, this negative effect is far less than the positive effects on SO_2_ emission reduction brought about by factors such as knowledge spillover, imitation learning and factor diffusion brought about by HSR opening in the neighboring cities. In other words, the positive impact of the SO_2_ reduction effect of the HSR on the neighboring cities is far greater than its negative impact. From the perspective of factor transfer, the emission reduction effect of HSR does not necessarily come at the expense of environmental pollution in neighboring cities. We also find that there will be more room for the positive spillover effect of HSR if relevant supporting measures for industrial transfer are adopted to actively address the emission problems of polluting enterprises with backward production capacity entering from neighboring cities.

### Analysis of heterogeneity

There are huge differences in resource endowments, geographic locations, and policy systems among cities in China, resulting in heterogeneity in the environmental effect of HSR. Hence, the differential impact of HSR opening on SO_2_ emissions reduction in terms of city tiering and income level is shown in Table 4. We use China’s 1–5 tier cities as the basis for classifying different city levels. We multiply the city level with HSR, as well as the *W*HSR, $W_{T,T}$ HSR, and $W_{NT,T}$ HSR variables that measure the spatial spillover effect, and brought these variables into the empirical model to evaluate the heterogeneous effect of HSR on SO_2_ emission in different city levels. The results are shown in columns (1), (2) of Table 4. In addition, we also multiplied the city’s per capita income level with the above variables, and brought their interaction terms into the empirical model to examine whether the SO_2_ emission reduction effect of HSR varies depending on the city’s per capita income level. The results are shown in columns (3), (4) of Table 4.

**Table 4:** Heterogeneity analysis of the environmental effects of HSR on SO_2_ emission reduction.

Variables	City-level heterogeneity		Income-level heterogeneity	
	SO_2_			
	(1)	(2)	(3)	(4)
HSR*City_type	− 0.043**	− 0.032*	0.406***	0.484***
	(0.018)	(0.018)	(0.108)	(0.098)
W*HSR*City_type	− 0.093***		− 0.577***	
	(0.033)		(0.154)	
${W}_{T,T}$*HSR*City_type		− 0.006*		− 0.077***
		(0.003)		(0.017)
${W}_{NT,T}$*HSR*City_type		− 0.025		− 0.066*
		(0.020)		(0.036)
rho	0.469***	0.466***	0.447***	0.438***
	(0.017)	(0.017)	(0.018)	(0.018)
R-squared	0.346	0.352	0.362	0.384
LR_Direct				
HSR_Xlevel	− 0.059***		0.504***	
	(0.019)		(0.107)	
${W}_{T,T}$*HSR*City_type		− 0.007**		− 0.083***
		(0.003)		(0.017)
${W}_{NT,T}$*HSR*City_type		− 0.026**		− 0.069*
		(0.010)		(0.037)
LR_Indirect				
HSR_City_type	− 0.203***		− 1.297***	
	(0.057)		(0.225)	
${W}_{T,T}$*HSR*City_type		− 0.005**		− 0.057***
		(0.003)		(0.011)
${W}_{NT,T}$*HSR*City_type		− 0.020		− 0.048*
		(0.018)		(0.026)
Control variables	YES	YES	YES	YES
Time Fixed	YES	YES	YES	YES
City Fixed	YES	YES	YES	YES
N	3135	3135	3135	3135

Note: Robust standard errors are in parentheses. The star mark represents its significance level, and ***, **, * indicate the significance levels at l%, 5%, and l0%, respectively.

The coefficients of $$HSR*City\_type$$ and $$W*HSR*City\_type$$ in column (1) are both statistically significantly negative, which indicates that the effect of HSR on SO2 emission reduction in local and neighboring cities increases with the cities’ tier (from the first-tier cities to the fifth-tier cities). In other words, compared with first- and second-tier cities with higher city levels, the lower the city level, the stronger the SO_2_ emission reduction effect of opening HSR on local and neighboring cities. Meanwhile, the coefficients of the interaction terms in both the direct and indirect effects of the decomposed SBM model are also significantly negative at the 1% level, which also confirms that the spatial spillover effects of the HSR on SO_2_ emission reduction, both exerted locally and in the neighboring areas, gradually increase with the decline of the city levels. Column (2) tests the heterogeneity of the decomposition of spatial spillover effects. The results show that the coefficient of $${W}_{T,T}$$*HSR*City_type is significantly negative, and the indirect effects of the SDM model decomposition also show significantly negative coefficients on the interaction terms, indicating that the spillover effect of HSR opening on SO_2_ emission reduction in neighboring HSR cities increases as the city level decreases. In the classification of China’s first- to fifth-tier cities, most of the first- or second-tier cities with relatively high urban levels are municipalities with regional political functions or provincial capital cities with higher administrative levels. These cities have better economic and policy resources, and most of the various high-quality production factors are concentrated here, which makes the industrial structure of these cities more reasonable and the production process more environmentally friendly. Therefore, the emission reduction effect of the opening of the HSR on SO_2_ is relatively limited. In particular, these cities are often the business card of cities in the province or even a larger region, and serve as benchmarks for other cities in all aspects of economy and environment. In order to achieve high-standard environmental goals, these cities may transfer their backward and high-pollution-emitting enterprises to promote further upgrading and renewal of industries in this region. However, those neighboring cities that have achieved transportation connections are usually the destinations to undertake the transfer of their backward production capacity, which also leads to the fact that the SO_2_ emission reduction effect of HSR on the neighboring HSR cities tends to be relatively stronger in the lower tier cities.

Columns (3) and (4) introduce interaction terms including urban per capita income into the model to further discuss how the SO_2_ emission reduction effect of HSR plays a heterogeneous role in cities with different income levels. The coefficient of HSR*City_type in column (3) is significantly positive at the 1% level, indicating that the local emission reduction effect of HSR weakens as the city’s per capita income level increases. This is also illustrated by the significantly positive coefficient on the interaction term in the direct effect of the decomposition of the SBM model. That is, HSR has a stronger emission reduction effect in low-income cities. This is because the HSR development not only brings production factors such as population, capital, and technology to low-income areas, but also promotes more diversified industrial development opportunities in this area. In particular, the HSR development in some extremely poor areas can reduce the dependence on energy extraction and the destruction of natural resources to maintain their livelihoods, and reduce SO_2_ pollution emissions. Moreover, the HSR opening in low-income areas is usually a breakthrough from scratch, and the marginal effect of social and economic development brought about by HSR is stronger. The coefficient of $W$ and the coefficient on the interaction term in the indirect effect are significantly negative at the 1% level, which shows that the spillover effect of HSR on SO_2_ emission reduction in neighboring cities increases with the increase in the city’s per capita income level. In other words, in cities with high-income levels, the spatial spillover effect of HSR on SO_2_ emission reduction in neighboring cities is stronger. Similarly, the decomposition effect of spatial spillover in column (4) shows that the coefficients of $W_{T,T}$*HSR*City_type and $W_{NT,T}$*HSR*City_type are both significantly negative, which shows that the SO_2_ abatement effect of HSR on these cities plays a stronger role in higher-income cities, regardless of whether they are neighboring HSR cities or neighboring cities without HSR. The reason may be that cities with high-income levels have more funds to attract high-tech talents and enterprises, and neighboring cities can usually benefit more from them through imitation, learning and knowledge spillover effects.

## Conclusions

SO_2_ emissions are the byproducts of economic development and have significant heterogeneity across regions in China. As one of the most important China’s modern means of transportation, HSR has effectively promoted the flow of production factors between regions, enabling cities where HSR has opened to achieve technological innovation and industrial structure restructuring, thereby changing the distribution pattern of SO_2_ emission. Our findings show that: Firstly, there is still an inter-regional imbalance in SO_2_ emissions in China city, the emissions of eastern coastal cities are significantly lower than those of central and western cities. In particular, those cities with serious SO_2_ emissions have been continuously concentrated in the southwest and northern. Secondly, HSR opening plays a significant inhibitory role in local SO_2_ emissions. Technological innovation and industrial structure upgrading are the internal urban accumulation mechanisms, and the cross-regional flow of production factors is the external urban interaction mechanism. Thirdly, HSR opening also plays a significant spatial spillover effect on SO_2_ emission reduction in neighboring cities. Moreover, the spatial spillover effect of neighboring cities without HSR is significantly greater than that of neighboring cities with HSR. Fourth, we find that the SO_2_ emissions reduction effect of HSR has heterogeneous characteristics at different cities’ tier and income levels. That is, the effect of HSR on local SO_2_ emission reduction is more obvious in cities with lower tiers and lower incomes. The spillover effect of HSR on neighboring cities is more obvious in lower-tier and higher-income cities, but the SO_2_ emission reduction effect of HSR on neighboring cities without HSR is still stronger in high-tier cities.

Based on the above conclusions, three policy recommendations are proposed. (1) Improving the regional HSR network from a balanced and open perspective. To achieve a balanced construction of HSR, relevant departments and policies should scientifically promote the extension of HSR to western cities, so as to promote the balance of high-speed rail networks in the eastern, central, and western regions, and gradually achieve full coverage of high-speed rail networks. Especially the high-speed rail network connectivity of non-provincial capital cities and small and medium-sized cities. Through the improvement of the accessibility of the transportation network, we can better play its media role, give full play to the energy-saving and emission reduction effect of HSR, coordinate regional green development, and maximize the positive impact of HSR on urban SO_2_ emission reduction. (2) Avoiding the old path of ‘pollution first, treatment later’ in the central and western regions. The HSR development has enhanced the mobility of production factors. Due to the increase in environmental protection thresholds and the shortage of production factors such as land and labor in the eastern region, many polluting enterprises have gradually moved from the east to the central and western regions. The governments of the central and western regions not only shoulder the heavy responsibility of developing the economy and narrowing the gap with the eastern regions, but also face the hard constraints of ecological environment protection. In order to prevent the emergence of an industrial development model at the cost of damaging the environment, an environmental access system should be established scientifically, and some polluting enterprises should be rationally absorbed according to the carrying capacity of the local environment. Meanwhile, establishing and improving the ecological environment assessment system, standardizing the clean production and operation, implementing ecological compensation measures in the undertaking areas, and promoting sustainable development. (3) Establishing an effective regional cooperation mechanism to enhance the promotion role of HSR development in terms of green, coordination and sharing. Making full use of the advantages of spatial location and the bonus of talent agglomeration brought about by the HSR development, and cultivating urban characteristic industries and new service industry growth points around the HSR. Cities with HSR will promote the upgrading of the industrial structure through the development of high-value-added service industries, thereby promoting the emission reduction effect of SO_2_. In addition, speeding up the construction of supporting infrastructure around the HSR station, building a green transportation system of ‘zero-distance transfer, seamless connection’, and fully releasing the spatial spillover effect of HSR SO_2_ emission reduction.

## Data Availability

The datasets analyzed during the current study are available from the corresponding author on reasonable request.

## References

[1] Wang T, Wang P, Theys N, Tong D, Hendrick F, Zhang Q, Van Roozendael M (2018). Spatial and temporal changes in SO_2_ regimes over China in the recent decade and the driving mechanism. Atmos. Chem. Phys..

[2] Chen Y, Ebenstein A, Greenstone M, Li H (2013). Evidence on the impact of sustained exposure to air pollution on life expectancy from China’s Huai River policy. Proc. Natl. Acad. Sci..

[3] Shi W, Du Y, Chang CH, Nguyen S, Wu J (2021). Spatial heterogeneity and economic driving factors of SO_2_ emissions in China: Evidence from an eigenvector based spatial filtering approach. Ecol.l Indic..

[4] Ling Z, Huang T, Zhao Y, Li J, Zhang X, Wang J, Lian L, Mao X, Gao H, Ma J (2017). OMI-measured increasing SO_2_ emissions due to energy industry expansion and relocation in northwestern China. Atmos. Chem. Phys..

[5] Sinha A, Bhattacharya J (2017). Estimation of environmental Kuznets curve for SO_2_ emission: A case of Indian cities. Ecol. Indic..

[6] Cheng Z, Li L, Liu J (2017). The emissions reduction effect and technical progress effect of environmental regulation policy tools. J. Clean. Prod..

[7] Fosten J, Morley B, Taylor T (2012). Dynamic misspecification in the environmental Kuznets curve: Evidence from CO_2_ and SO_2_ emissions in the United Kingdom. Ecol. Econ..

[8] He Z, Shi X, Wang X, Xu Y (2017). Urbanisation and the geographic concentration of industrial SO_2_ emissions in China. Urban Stud..

[9] Soytas U, Sari R, Ewing BT (2007). Energy consumption, income, and carbon emissions in the United States. Ecol. Econ..

[10] Wang Y, Han R, Kubota J (2016). Is there an environmental Kuznets curve for SO_2_ emissions? A semi-parametric panel data analysis for China. Renew. Sustain. Energy Rev..

[11] Yang M, Ma T, Sun C (2018). Evaluating the impact of urban traffic investment on SO_2_ emissions in China cities. Energy Policy.

[12] Cao J, Liu XC, Wang Y, Li Q (2013). Accessibility impacts of China’s high-speed rail network. J. Transp. Geogr..

[13] Vickerman R (2015). High-speed rail and regional development: The case of intermediate stations. J. Transport Geogr..

[14] Zhou Z, Zhang A (2021). High-speed rail and industrial developments: Evidence from house prices and city-level GDP in China. Transport. Res. Part A: Policy Pract..

[15] Åkerman J (2011). The role of high-speed rail in mitigating climate change—the Swedish case Europabanan from a life cycle perspective. Transp. Res. Part D: Transp. Environ..

[16] Yang X, Lin S, Li Y, He M (2019). Can high-speed rail reduce environmental pollution? Evidence from China. J. Clean. Prod..

[17] Li H, Guo H (2021). Spatial spillovers of pollution via high-speed rail network in China. Transport Policy.

[18] Li Q, Dong A, Zhang B (2022). Impact of the opening of high-speed rail on environmental pollution in the Yangtze River Economic Belt: Promoting or inhibiting?. Int. J. Environ. Sci. Technol.

[19] Lin J, Li H, Huang W, Xu W, Cheng S (2019). A carbon footprint of high-speed railways in China: A case study of the beijing-Shanghai line. J. Ind. Ecol..

[20] Albalate, D. & Bel, G. The economics and politics of high-speed rail: Lessons from experiences abroad. Rowman and Littlefield Publishers (Lexington Books), Lanham, Ma. https://rowman.com/ISBN/9780739171233 (2012).

[21] Shaw SL, Fang Z, Lu S, Tao R (2014). Impacts of high speed rail on railroad network accessibility in China. J. Transport Geogr..

[22] Guirao B, Campa JL, Casado-Sanz N (2018). Labour mobility between cities and metropolitan integration: The role of high speed rail commuting in Spain. Cities.

[23] Fan X, Xu Y, Nan Y, Li B, Cai H (2020). Impacts of high-speed railway on the industrial pollution emissions in China: Evidence from multi-period difference-in-differences models. Kybernetes.

[24] Gao Y, Zheng J, Wang X (2022). Does high-speed rail reduce environmental pollution? Establishment-level evidence from China. Socio-Econ. Plan. Sci..

[25] Liu S, Zhang Y, Cai J (2023). Operation of high-speed rail and reduction of corporate pollution: Evidence from China. Environ. Sci. Pollut. Res..

[26] Spulber, D. F. *Global competitive strategy* (Cambridge University Press, 2007). https://EconPapers.repec.org/RePEc:cup:cbooks:9780521367981.

[27] Qin Y (2017). No county left behind?’The distributional impact of high-speed rail upgrades in China. J. Econ. Geogr..

[28] Zhao X, Yin H (2011). Industrial relocation and energy consumption: Evidence from China. Energy Policy.

[29] Jia R, Shao S, Yang L (2021). High-speed rail and CO_2_ emissions in urban China: A spatial difference-in-differences approach. Energy Econ..

[30] Liu Q, Li H, Shang WL, Wang K (2022). Spatio-temporal distribution of Chinese cities’ air quality and the impact of high-speed rail. Renew. Sustain. Energy Rev..

[31] Ferman B (2023). Inference in difference-in-differences: How much should we trust in independent clusters?. J. Appl. Econometr..

[32] Wu T, Lin S, Wang J, Yan N (2023). High-speed rail and city’s carbon productivity in China: A spatial difference-in-differences approach. Environ. Sci. Pollut. Res..

[33] Sun X, Yan S, Liu T, Wu J (2020). High-speed rail development and urban environmental efficiency in China: A city-level examination. Transport. Res. Part D: Transport Env..

[34] Wang F, Shan J, Liu J, Fan W, Yan B, Zhao H, Luo S (2022). How does high-speed rail construction affect air pollutant emissions? Evidence from the Yangtze River Delta Urban Agglomeration in China. J. Clean. Prod..

[35] Yan L, Tu M, Chagas AL, Tai L (2022). The impact of high-speed railway on labor spatial misallocation—Based on spatial difference-in-differences analysis. Transport. Res. Part A: Policy Practice.

[36] Gibbons S, Machin S (2005). Valuing rail access using transport innovations. J. Urban Econ..

[37] Catalini, C., Fons-Rosen, C. & Gaule, P. Did cheaper flights change the direction of science? In *CEPR Discussion Paper DP11252*. https://ssrn.com/abstract=2774366 (2016).

[38] Levinson A (2009). Technology, international trade, and pollution from US manufacturing. Am. Econ. Rev..

[39] Huang Y, Wang Y (2020). How does high-speed railway affect green innovation efficiency? A perspective of innovation factor mobility. J. Clean. Prod..

[40] Li Z, Xu H (2018). High-speed railroads and economic geography: Evidence from Japan. J. Reg. Sci..

[41] Nie L, Zhang Z (2023). Is high-speed rail heading towards a low-carbon industry? Evidence from a quasi-natural experiment in China. Resourc. Energy Econ..

[42] Zhao M, Liu X, Derudder B, Zhong Y, Shen W (2015). Mapping producer services networks in mainland Chinese cities. Urban Stud..

[43] Dinda S (2004). Environmental Kuznets curve hypothesis: A survey. Ecol. Econ..

[44] Chen Z, Xue J, Rose AZ, Haynes KE (2016). The impact of high-speed rail investment on economic and environmental change in China: A dynamic CGE analysis. Transport. Res. Part A: Policy Pract..

[45] Ureña JM, Menerault P, Garmendia M (2009). The high-speed rail challenge for big intermediate cities: A national, regional and local perspective. Cities.

[46] Huang Y, Jiang C, Wang K, Xiao Y, Zhang A (2021). Public-private partnership in high-speed rail financing: Case of uncertain regional economic spillovers in China. Transport policy.

[47] Banerjee A, Duflo E, Qian N (2020). On the road: Access to transportation infrastructure and economic growth in China. J. Dev. Econ..

[48] Huang G, Zhang J, Yu J, Shi X (2020). Impact of transportation infrastructure on industrial pollution in Chinese cities: A spatial econometric analysis. Energy Econ..

[49] Givoni M (2006). Development and impact of the modern high-speed train: A review. Transport Rev..

[50] Lin Y (2017). Travel costs and urban specialization patterns: Evidence from China’s high speed railway system. J. Urban Econ..

[51] Zheng S, Kahn ME (2013). China’s bullet trains facilitate market integration and mitigate the cost of megacity growth. Proc. Natl. Acad. Sci..

[52] Garmendia M, de Ureña JM, Ribalaygua C, Leal J, Coronado JM (2008). Urban residential development in isolated small cities that are partially integrated in metropolitan areas by high speed train. Eur. Urban Reg. Stud..

[53] Baldwin RE, Okubo T (2005). Heterogeneous firms, agglomeration and economic geography: Spatial selection and sorting. J. Econ. Geogr..

[54] Shao S, Chen Y, Li K, Yang L (2019). Market segmentation and urban CO_2_ emissions in China: Evidence from the Yangtze River Delta region. J. Environ. Manage..

[55] Zhang YJ, Da YB (2015). The decomposition of energy-related carbon emission and its decoupling with economic growth in China. Renew. Sustain. Energy Rev..

[56] Liu X, Zhang X (2021). Industrial agglomeration, technological innovation and carbon productivity: Evidence from China. Resourc. Conserv. Recycl..

[57] Sun H, Edziah BK, Kporsu AK, Sarkodie SA, Taghizadeh-Hesary F (2021). Energy efficiency: The role of technological innovation and knowledge spillover. Technol. Forecast. Soc. Change.

[58] Wang Y, Cao G, Yan Y, Wang J (2022). Does high-speed rail stimulate cross-city technological innovation collaboration? Evidence from China. Transport. Policy.

[59] Gu R, Li C, Li D, Yang Y, Gu S (2022). The impact of rationalization and upgrading of industrial structure on carbon emissions in the Beijing-Tianjin-Hebei urban agglomeration. Int. J. Environ. Res. Public Health.

[60] Yang J, Liu C, Liu K (2023). Land marketization and industrial restructuring in China. Land Use Policy.

[61] Zhao L, Zhang X, Zhao F (2020). Evaluating the impact of high-speed rail on county-level air quality in China. Transport. Res. part D: Transport Env..

[62] Jiao J, Wang J, Zhang F, Jin F, Liu W (2020). Roles of accessibility, connectivity and spatial interdependence in realizing the economic impact of high-speed rail: Evidence from China. Transport Policy.

[63] Zheng S, Zhang X, Sun W, Wang J (2019). The effect of a new subway line on local air quality: A case study in Changsha. Transport. Res. part D: Transport Env..

[64] Huang Y, Xu W (2021). Spatial and temporal heterogeneity of the impact of high-speed railway on urban economy: Empirical study of Chinese cities. J. Transport Geogr..

[65] Chagas AL, Azzoni CR, Almeida AN (2016). A spatial difference-in-differences analysis of the impact of sugarcane production on respiratory diseases. Reg. Sci. Urban Econ..

[66] Baker AC, Larcker DF, Wang CC (2022). How much should we trust staggered difference-in-differences estimates?. J. Financ. Econ..

[67] Callaway B, Sant’Anna PH (2021). Difference-in-differences with multiple time periods. J. Econometr..

[68] De Chaisemartin C, d’Haultfoeuille X (2020). Two-way fixed effects estimators with heterogeneous treatment effects. Am. Econ. Rev..

[69] Goodman-Bacon A (2021). Difference-in-differences with variation in treatment timing. J. Econometr..

[70] De Chaisemartin C, d’Haultfoeuille X (2023). Two-way fixed effects and differences-in-differences with heterogeneous treatment effects: A survey. Econometr. J..

[71] Faber B (2014). Trade integration, market size, and industrialization: Evidence from China’s National Trunk Highway System. Rev. Econ. Stud..

[72] Baum-Snow N, Brandt L, Henderson JV, Turner MA, Zhang Q (2017). Roads, railroads, and decentralization of Chinese cities. Rev. Econ. Stat..

[73] Duranton G, Turner MA (2012). Urban growth and transportation. Rev. Econ. Stud..

[74] Duflo E, Pande R (2007). Dams. Q. J. Econ..

[75] LeSage, J. P. & Pace, R. K. Spatial econometric models. In *Handbook of Applied Spatial Analysis: Software Tools, Methods and Applications* (Springer, 2009). 10.1007/978-3-642-03647-7_18.

